# Genomic profiling in Bulgarian women with ovarian cancer: a dual-sample NGS approach

**DOI:** 10.1186/s13053-026-00336-z

**Published:** 2026-03-25

**Authors:** Zornitsa Kamburova, Savelina Popovska, Chavdar Tsvetkov

**Affiliations:** 1 Department of Medical Genetics, Faculty of Pharmacy, Medical University, Pleven, 1 “St Kliment Ohridski” str, Pleven, 5800 Bulgaria; 2 Centre of Competence in Personalized Medicine, 3D and Telemedicine, Robotic Assisted and Minimally Invasive Surgery – “Leonardo da Vinci”, Pleven, 5800 Bulgaria; 3 Department of General and Clinical Pathology, Faculty of Medicine, Medical University, Pleven, 5800 Bulgaria; 4 Department of Midwifery Care, Faculty of Health Care, Medical University, Pleven, 5800 Bulgaria

**Keywords:** Ovarian cancer, Next‑generation sequencing, Germline, Somatic, *BRCA1*, *TP53*, Homologous recombination, Bulgarian cohort

## Abstract

**Background:**

Ovarian cancer is the deadliest gynecologic malignancy largely due to late diagnosis. The present study aims to provide a comprehensive assessment of clinically relevant and potentially actionable germline and somatic pathways in ovarian cancer through parallel analysis of inherited susceptibility and tumor-specific genomic alterations, within the constraints of targeted NGS panel design.

**Methods:**

We analyzed 33 Bulgarian women with histologically confirmed ovarian cancer. Next‑generation sequencing (NGS) was performed on germline DNA from peripheral blood and tumor DNA from FFPE tissue. Variants were described per HGVS, classified by ACMG/AMP (germline) and AMP/ASCO/CAP (somatic), and reviewed in ClinVar/COSMIC. Descriptive statistics were generated in Python.

**Results:**

All pathogenic and likely pathogenic germline and somatic variants are summarized in comprehensive tables. Somatic pathogenic TP53 variants were identified in 27/33 patients (81.8%). Germline pathogenic/likely pathogenic variants were detected in 9/33 (27.3%), most commonly in *BRCA1* (3/33; 9.1%), with single cases in *ATM*,* RAD51D*,* NBN*,* FANCL* and *WRN*. One patient fulfilled criteria for multi‑locus inherited neoplasia alleles syndrome (MINAS). High‑grade serous ovarian histological subtype predominated (75.8%).

**Conclusions:**

Dual-sample NGS enables an integrated evaluation of hereditary risk and tumor-associated actionable alterations in ovarian cancer. While comprehensive interrogation of all therapeutic pathways is limited by panel scope, this approach supports clinically meaningful stratification and highlights areas requiring expanded molecular testing.

## Background

Ovarian cancer (OC) remains one of the leading causes of cancer-related death among women worldwide, with an estimated 313,000 new cases reported annually according to GLOBOCAN 2020 [1]. The majority of cases—particularly high-grade serous carcinoma (HGSC)—harbor *TP53* somatic mutations in nearly all instances, along with frequent defects in homologous recombination repair, most often associated with *BRCA1* and *BRCA2* mutations [2, 3].

Germline *BRCA1/2* variants markedly increase a woman’s lifetime risk of developing the disease and serve as strong predictors of responsiveness to platinum-based chemotherapy and PARP inhibitors [4, 5]. Recent studies, however, highlight a broader spectrum of germline alterations involving other genes such *as RAD51C*,* RAD51D*,* ATM*,* NBN*, and *FANCL* [6, 8]. Somatic genomic profiling further expands the therapeutic landscape, revealing actionable targets in genes including *PIK3CA*,* KRAS*,* ERBB2*, and *AKT1* [9, 11].

The present study integrates germline and tumor genomic data from Bulgarian patients with ovarian cancer with the aim of providing a comprehensive assessment of actionable molecular pathways relevant to hereditary risk stratification and tumor biology. By applying a dual-sample NGS approach, the study seeks to characterize both inherited susceptibility and somatic driver alterations, while explicitly acknowledging the limitations imposed by targeted panel design on the full evaluation of all therapeutic pathways.

## Methods

### Patients and study design

This study included 33 unrelated Bulgarian women diagnosed with epithelial ovarian or fallopian tube carcinoma between 2017 and 2024. Peripheral blood samples for germline DNA extraction and formalin-fixed paraffin-embedded (FFPE) tumor tissue blocks were collected from each participant. Genomic DNA extracted from both peripheral blood and FFPE tumor tissues was quantified using the Qubit fluorometric system before library preparation. All patients provided written informed consent prior to enrollment.

### Germline DNA extraction and sequencing

Genomic DNA was isolated from peripheral blood using the MagCore^®^ Genomic DNA Whole Blood Kit (MagCore) following the manufacturer’s instructions. Targeted next-generation sequencing (NGS) was performed using the TruSight Hereditary Cancer Panel (Illumina Inc.), which captures exons and exon–intron boundaries of 113 genes associated with hereditary cancer syndromes. Library preparation was performed according to the manufacturer’s protocol, and qualified libraries were sequenced on the Illumina MiSeq platform using a 2 × 150 bp configuration. Sequence reads were aligned to the human reference genome (hg19).

### Tumor DNA extraction and sequencing

All FFPE samples were reviewed by an experienced pathologist and tumor-rich areas were selected prior to sequencing. Tumor DNA was extracted from FFPE tissue using the QIAamp DNA FFPE Tissue Kit (QIAGEN, Germany) according to the manufacturer’s protocol. DNA quantity and quality were assessed with a Qubit 2.0 Fluorometer (Thermo Fisher Scientific) using the Qubit dsDNA HS Assay Kit. Targeted NGS of tumor DNA was carried out with the TruSight Tumor 15 Panel (Illumina Inc.), covering 250 amplicons across 15 solid-tumor–associated genes (***AKT1***,*** BRAF***,*** EGFR***,*** ERBB2***,*** FOXL2***,*** GNA11***,*** GNAQ***,*** KIT***,*** KRAS***,*** MET***,*** NRAS***,*** PDGFRA***,*** PIK3CA***,*** RET***). Libraries were sequenced on the Illumina MiSeq (2 × 150 bp) and reads were aligned to hg19.

### Variant calling and classification

Sequencing data (gVCF files) from both germline and tumor samples were analyzed using Franklin (Genoox). Custom filters were applied, requiring a minimum read depth of 20× for germline variants and 500× for somatic variants, and excluding synonymous changes. Germline variants were classified according to the five-tier ACMG/AMP framework: Pathogenic (P), Likely Pathogenic (LP), Variant of Uncertain Significance (VUS), Likely Benign (LB), and Benign (B) [7, 12]. Automated annotations were manually reviewed against ClinVar, dbSNP, and Ensembl databases. Somatic variants were categorized according to AMP/ASCO/CAP guidelines: **Tier I** – Variants of strong clinical significance; **Tier II** – Variants of potential clinical significance; **Tier III** – Variants of uncertain significance; **Tier IV** – Benign or likely benign variants. All somatic annotations were manually verified in ClinVar, COSMIC, and OncoKB. Variant allele frequencies (VAF%) were recorded.

### Statistical analysis

Descriptive statistics and visualizations (including distribution plots and heatmaps) were generated in Python.

## Results

### Patient characteristics

The study cohort consisted of 33 women diagnosed with epithelial ovarian or fallopian tube carcinoma. The median age at diagnosis was 59.2 years (range: 20–83). High-grade serous carcinoma (HGSC) was the predominant histological subtype, observed in 69.7% (*n* = 23/33) of cases, followed by low-grade serous carcinoma (LGSC) – 15.2% (*n* = 5/33); endometrioid carcinoma (6.1%; *n* = 2/33) and other subtypes, each accounting for less than 5%. Most tumors were poorly differentiated: Grade 3 in 75.8% of patients, Grade 2 in 12.1%, and Grade 1 in 12.1%. Bilateral tumor localization was observed in 3 patients (9%). Positive family history for cancer was reported in 8 patients (24.2%), indicating potential hereditary predisposition. Baseline clinical, demographic, and histopathological characteristics of the study cohort are summarized in Table 1, including age at diagnosis, tumor subtype and grade, bilaterality, and family history of cancer.

**Table 1:** Baseline clinical and histological characteristics

Feature	Value
N (patients)	33
Median age	59.2 y (range 20–83)
Histology	HGSC 69.7%; LGSC 15.2%, endometrioid 6,1%; others < 5%
Tumor grade	G3 75.8%; G2 12,1%; G1 12,1%
Bilateral tumors	9% (*n* = 3/33)
Family history of cancer	24.2% (*n* = 8/33)

### Germline findings

Pathogenic or likely pathogenic germline variants were detected in **9 patients (27.3%)**. The most frequently altered gene was ***BRCA1***, identified in **3 patients (11%)**. Other genes with germline variants included ***ATM***, ***RAD51D***, ***NBN***, ***FANCL***, and ***WRN***, each detected in one patient. In one of the patient we detected MINAS (*TP53* and *FANCE*). All germline variants are presented in Table 2. These findings highlight the heterogeneity of germline alterations in ovarian cancer patients and underscore the importance of comprehensive genetic testing beyond *BRCA1/2*.

**Table 2:** Pathogenic and likely pathogenic germline variants detected in hereditary cancer genes

Gene	Transcript (RefSeq)	Nucleotide change (cDNA)	Protein change	rs number	Variant type	Exon	ACMG classification	Associated cancer risk / syndrome
ATM	NM_000051.3	c.8147T > C	p.(Val2716Ala)	rs56128736	Missense	55/63	Likely pathogenic	Breast, ovarian, pancreatic cancer
BRCA1	NM_007294.3	c.5266dup	p.(Gln1756ProfsTer74)	rs80357906	Frameshift	19/23	Pathogenic	Hereditary breast and ovarian cancer (HBOC)
BRCA1	NM_007294.3	c.5533dup	p.(Tyr1845LeufsTer35)	*NA (ClinVar-reported variant)*	Frameshift	23/23	Pathogenic	HBOC
BRCA1	NM_007294.3	c.181T > G	p.(Cys61Gly)	rs28897672	Missense	4/23	Pathogenic	HBOC
FANCL	NM_001114636.1	c.1048_1051del	p.(Gln350fs)	*NA (ClinVar-reported variant)*	Frameshift / stop gained	14/14	Pathogenic	Fanconi anemia pathway; predisposition to cancer
TP53	NM_000546.5	c.1148_1149del	p.(Leu383HisfsTer8)	*NA (ClinVar-reported variant)*	Frameshift	11/11	Pathogenic	Li-Fraumeni syndrome, multiple cancers
FANCE	NM_021922.2	c.1239dup	p.(Pro414SerfsTer54)	*NA (ClinVar-reported variant)*	Frameshift / splice	7/10	Pathogenic	Fanconi anemia pathway
NBN	NM_002485.4	c.2140 C > T	p.(Arg714Ter)	rs587781298	Stop gained	14/16	Pathogenic	Nijmegen breakage syndrome; breast/ovarian cancer risk
RAD51D	NM_002878.3	c.803G > A	p.(Trp268Ter)	rs879254418	Stop gained	9/10	Pathogenic	Ovarian cancer predisposition
WRN	NM_000553.4	c.1105 C > T	p.(Arg369Ter)	rs121908446	Stop gained	9/35	Pathogenic	Werner syndrome; cancer predisposition

### Gene-histology distribution

**Fig. 1:**
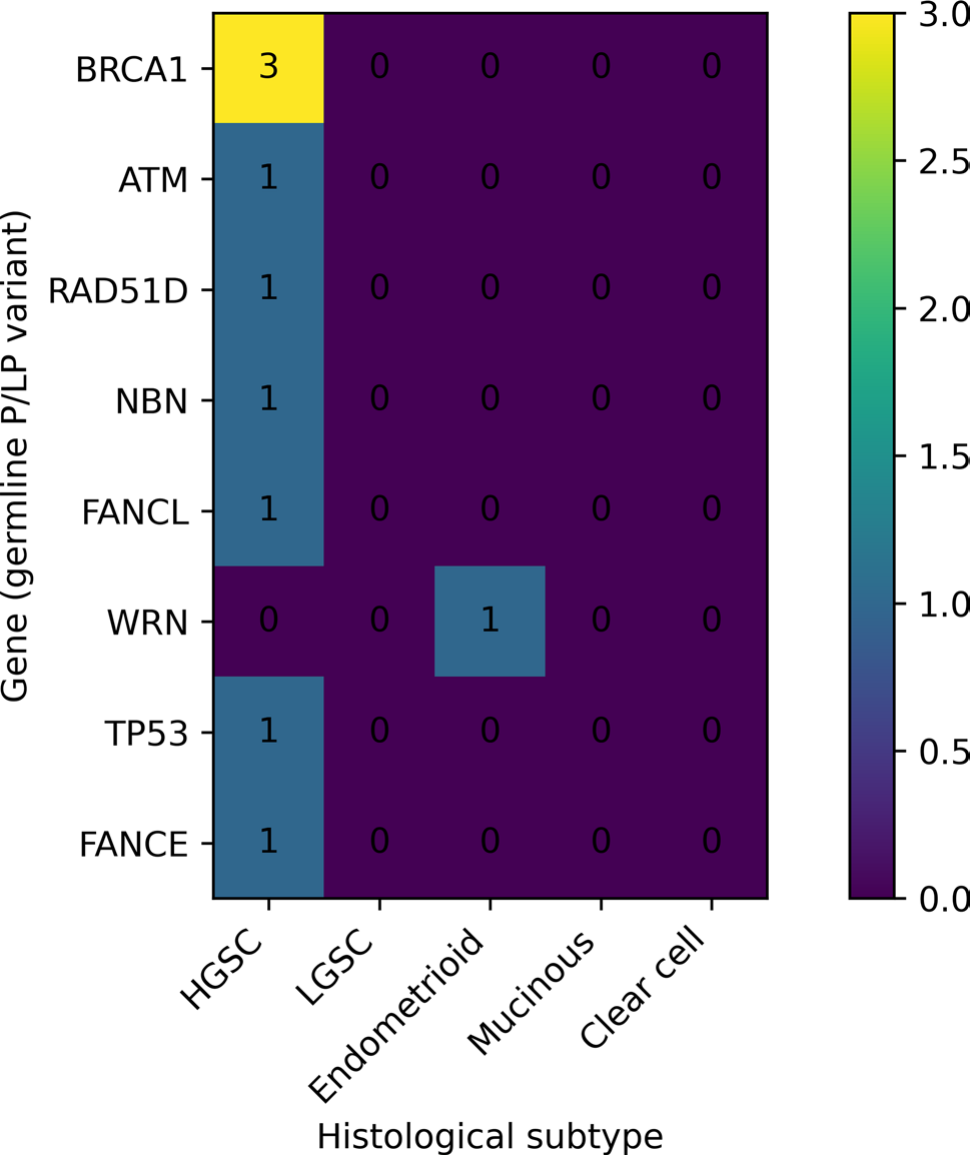
Heatmap of the distribution of pathogenic (P) and likely pathogenic (LP) germline variants across histological subtypes of ovarian carcinoma

*BRCA1* variants were predominantly detected in high-grade serous carcinomas. Other genes such as *ATM*,* FANCL*, and *RAD51D* also showed exclusive association with serous histology. Heatmap of the distribution of pathogenic (P) and likely pathogenic (LP) germline variants according to histological subtype is presented on Figure 1.This visualization highlights potential genotype–phenotype correlations that may inform individualized risk assessment and tailored therapy.

### Age distribution of pathogenic germline variants

**Fig. 2:**
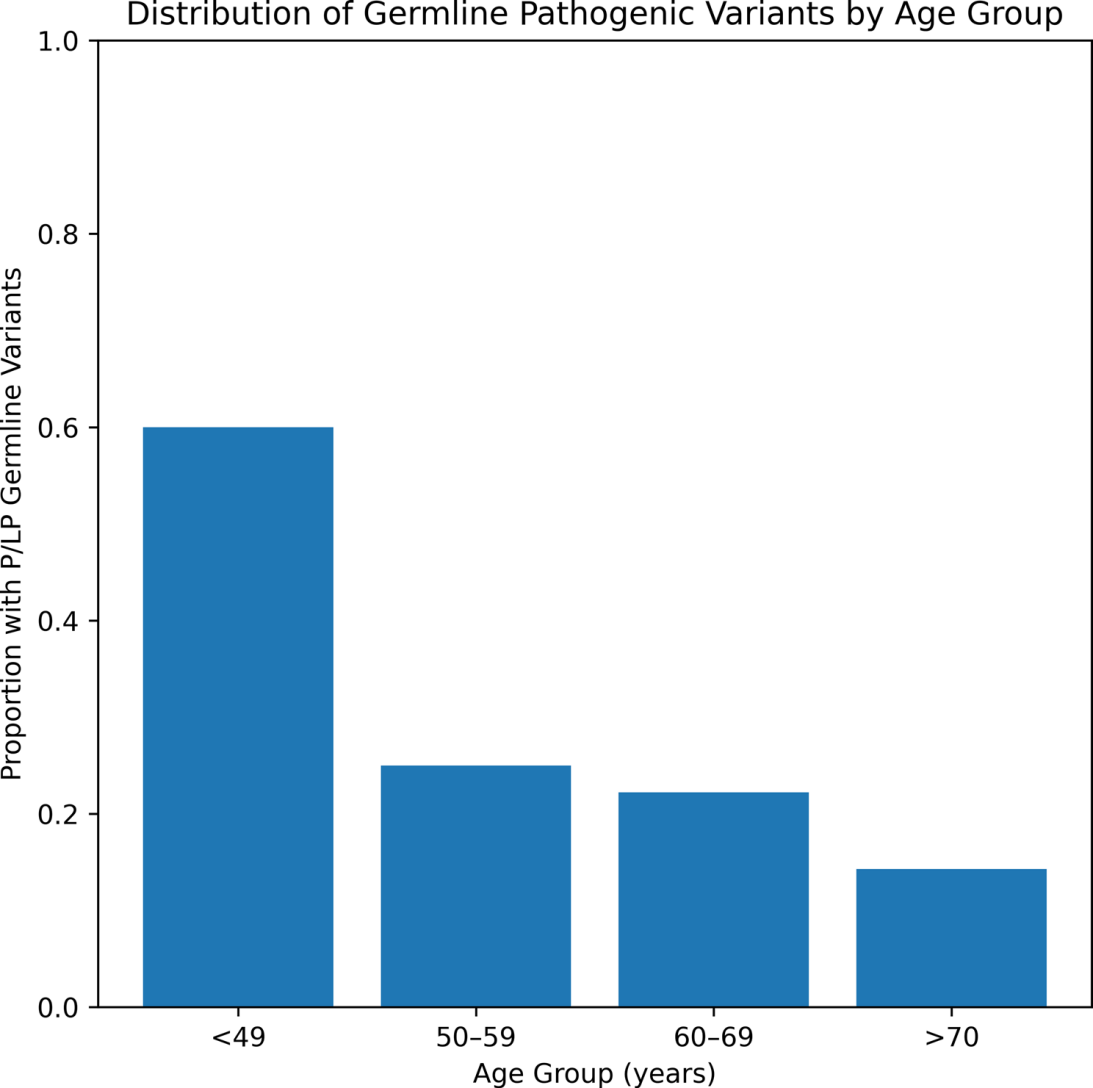
Distribution of germline pathogenic/likely pathogenic variants according to age group at diagnosis

The distribution of pathogenic and likely pathogenic variants was analyzed by age group. Most variants were identified in patients aged 50–69 years, with a peak in the 50–59 and 60–69 decades. The Fig. 2 illustrates the proportion of patients carrying pathogenic or likely pathogenic (P/LP) germline variants across predefined age groups at diagnosis. The highest relative frequency was observed in patients younger than 49 years (3/5; 60%). In the 50–59 age group, 3 of 12 women (25%) harbored P/LP germline variants. Among patients aged 60–69 years, 2 of 9 (22.2%) were variant-positive, while in those over 70 years, 1 of 7 (14.3%) carried a P/LP germline variant.

### Somatic findings

**Fig. 3:**
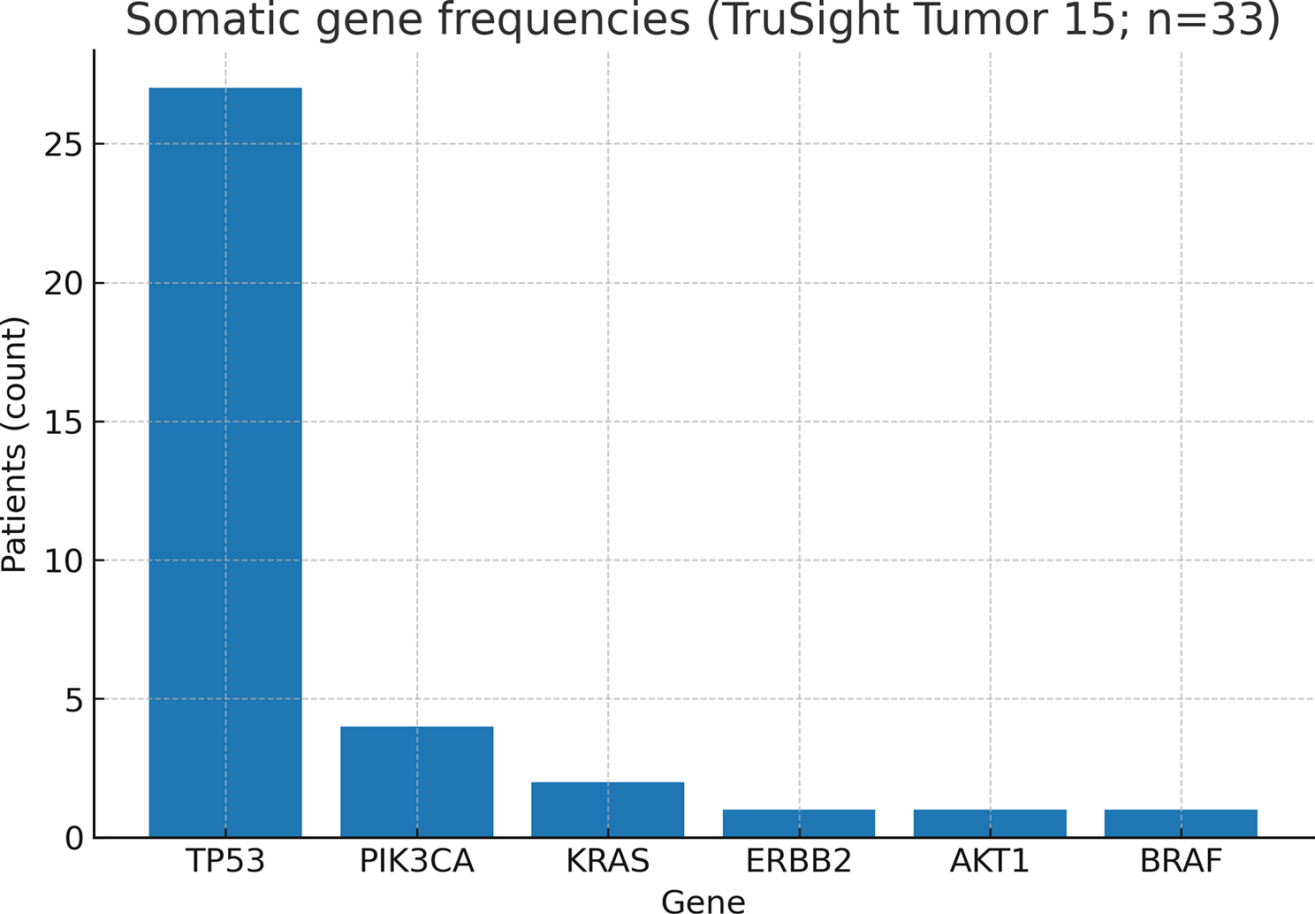
Frequency of top somatic genes across the cohort (bar plot)

**Fig. 4:**
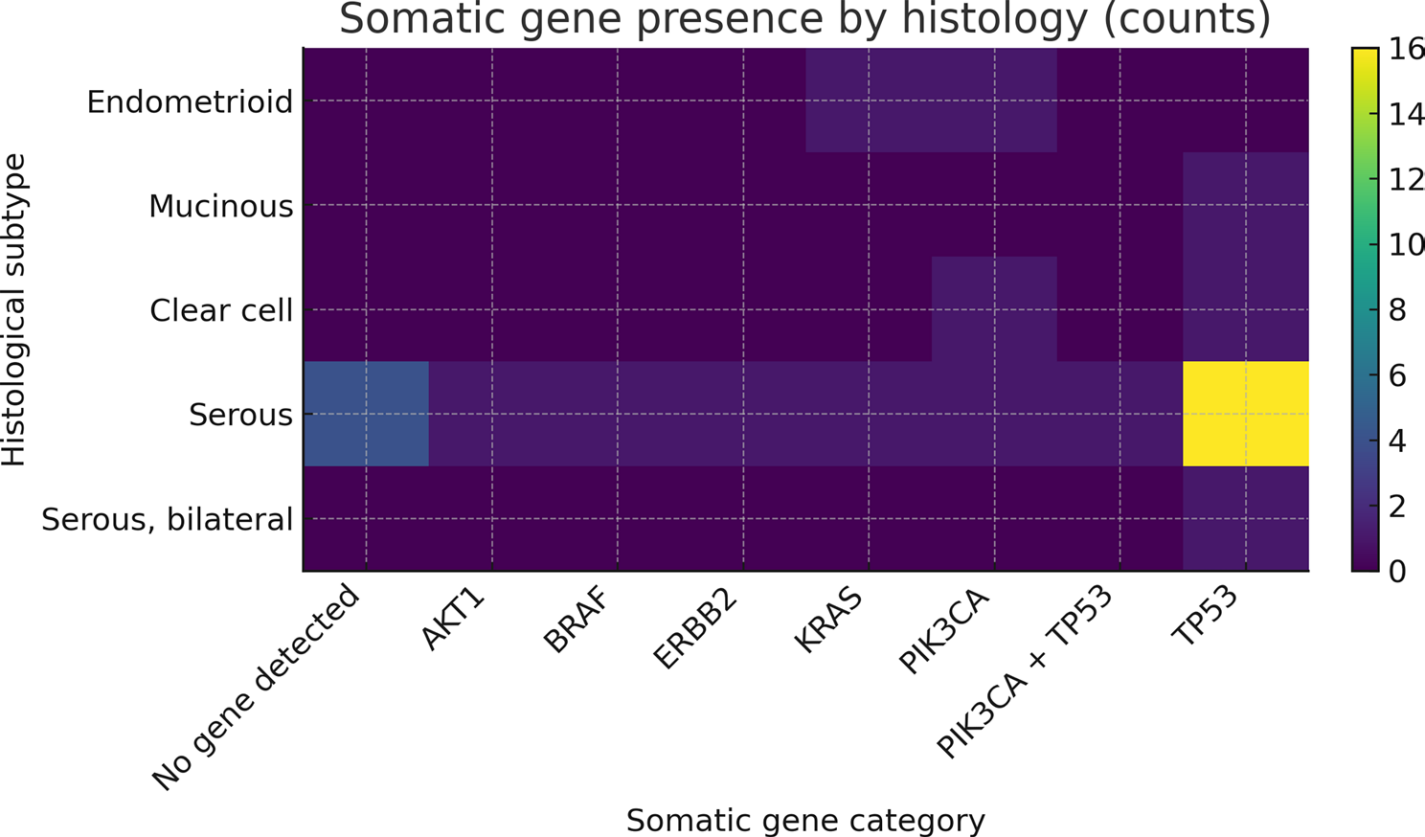
Somatic gene presence heatmap across histologies (top 15 genes)

Somatic alterations (FFPE) are summarized in Tables 3 and 4; Figs. 3 and 4; most frequent genes included *TP53* (*n* = 21), *PIK3CA* (*n* = 4), *KRAS* (*n* = 2), *BRAF* (*n* = 1).

**Table 3:** Somatic gene presence by histological subtype (TruSight Tumor 15 panel)

Histological type	No gene variant detected	AKT1	BRAF	ERBB2	KRAS	PIK3CA	PIK3CA + TP53	TP53
Endometrioid	0	0	0	0	1	1	0	0
Mucinous	0	0	0	0	0	0	0	1
Clear cell	0	0	0	0	0	1	0	1
Serous	4	1	1	1	1	1	1	16
Serous, bilateral	0	0	0	0	0	0	0	1

Somatic *TP53* pathogenic variants were identified in 21 patients (63.6%), with variant allele frequencies (VAF) ranging from 15% to 97%. Variants in other oncogenes included *PIK3CA* (4 patients, 12.1%), *KRAS* (2 patients), and *ERBB2*,* AKT1*,* BRAF*—each found in one patient.

Co-occurrence of *TP53* mutations with another actionable somatic variant (in *PIK3CA*) was observed in 1 patient (3%). These co-alterations may have therapeutic implications and highlight the molecular complexity of ovarian cancer.

**Table 4:** Pathogenic and likely pathogenic somatic variants detected in ovarian cancer patients

Gene	Transcript (RefSeq)	Nucleotide change (cDNA)	Protein change	Variant type	Exon	ACMG classification	Associated cancer risk / syndrome
*AKT1*	NM_001382430.1	c.49G > A	p.(Glu17Lys)	Missense	2	Pathogenic	Breast, endometrial cancers
*BRAF*	NM_004333.6	c.1799T > A	p.(Val600Glu)	Missense	15	Pathogenic	Melanoma, colorectal, thyroid, ovarian cancer
*ERBB2*	NM_004448.4	c.2313_2324dup	p.(Tyr772_Ala775dup)	In-frame duplication	20	Pathogenic	Breast, gastric cancers
*KRAS*	NM_004985.5	c.35G > T / c.35G > C	p.(Gly12Val) / p.(Gly12Ala)	Missense	2	Pathogenic	Colorectal, lung, pancreatic cancer
*KRAS*	NM_004985.5	c.37G > T	p.(Gly13Cys)	Missense	2	Pathogenic	Colorectal, pancreatic cancers
*PIK3CA*	NM_006218.4	c.1624G > A	p.(Glu542Lys)	Missense	9	Pathogenic	Breast, endometrial cancers
*PIK3CA*	NM_006218.4	c.1633G > A	p.(Glu545Lys)	Missense	9	Pathogenic	Breast, endometrial, colorectal cancers
*PIK3CA*	NM_006218.4	c.3139 C > T	p.(His1047Tyr)	Missense	20	Pathogenic	Multiple cancers
*TP53*	NM_000546.6	c.380 C > T	p.(Ser127Phe)	Missense	5	Likely pathogenic	Cancer predisposition
*TP53*	NM_000546.6	c.394 A > C	p.(Lys132Gln)	Missense	5	Likely pathogenic	Cancer predisposition
*TP53*	NM_000546.6	c.395 A > G	p.(Lys132Arg)	Missense	5	Likely pathogenic	Cancer predisposition
*TP53*	NM_000546.6	c.422G > A	p.(Cys141Tyr)	Missense	5	Pathogenic	Cancer predisposition
*TP53*	NM_000546.6	c.452 C > G	p.(Pro151Arg)	Missense	5	Likely pathogenic	Cancer predisposition
*TP53*	NM_000546.6	c.532del / c.839G > C	p.(His178ThrfsTer69) / p.(Arg280Thr)	Frameshift + missense	5 / 8	Pathogenic	Cancer predisposition
*TP53*	NM_000546.6	c.535 C > G / c.1148_1149del	p.(His179Asp) / p.(Leu383HisfsTer8)	Missense + frameshift	5 / 11	Pathogenic	Cancer predisposition
*TP53*	NM_000546.6	c.535 C > T / c.722 C > G	p.(His179Tyr) / p.(Ser241Cys)	Missense	5 / 7	Pathogenic	Cancer predisposition
*TP53*	NM_000546.6	c.536 A > G	p.(His179Arg)	Missense	5	Pathogenic	Cancer predisposition
*TP53*	NM_000546.6	c.659 A > G	p.(Tyr220Cys)	Missense	6	Pathogenic	Cancer predisposition
*TP53*	NM_000546.6	c.711_719dup	p.(Asn239_Ser240insArgCysAsn)	In-frame duplication	7	VUS / Likely pathogenic	Cancer predisposition
*TP53*	NM_000546.6	c.730G > A	p.(Gly244Ser)	Missense	7	Pathogenic	Cancer predisposition
*TP53*	NM_000546.6	c.742 C > T	p.(Arg248Trp)	Missense	7	Pathogenic	Cancer predisposition
*TP53*	NM_000546.6	c.743G > A	p.(Arg248Gln)	Missense	7	Pathogenic	Li-Fraumeni syndrome, multiple cancers
*TP53*	NM_000546.6	c.722 C > T	p.(Ser241Phe)	Missense	7	Pathogenic	Cancer predisposition
*TP53*	NM_000546.6	c.796G > A	p.(Gly266Arg)	Missense	8	Pathogenic	Cancer predisposition
*TP53*	NM_000546.6	c.844 C > T	p.(Arg282Trp)	Missense	8	Pathogenic	Cancer predisposition
*TP53*	NM_000546.6	c.856G > A	p.(Glu286Lys)	Missense	8	Pathogenic	Cancer predisposition
*TP53*	NM_000546.6	c.916 C > T	p.(Arg306Ter)	Stop gained	8	Pathogenic	Cancer predisposition
*TP53*	NM_000546.6	c.920-1G > C	Splice acceptor	Splice site	9	Pathogenic	Cancer predisposition

### Stratified analysis by histological subtype and serous tumor grade

To investigate histotype-specific and grade-dependent patterns of germline and somatic variation, patients were stratified according to histological subtype of ovarian carcinoma, followed by a secondary stratification of serous tumors into high-grade and low-grade categories based on histological grade.

### Stratification by histological subtype

The cohort consisted predominantly of serous carcinomas (*n* = 27), followed by endometrioid (*n* = 3), clear cell (*n* = 2), mucinous (*n* = 1), and one case with unavailable histological classification.

### Germline pathogenic/likely pathogenic variants across histological subtypes

Overall, germline pathogenic or likely pathogenic (P/LP) variants were identified in 9 of 33 patients (27.3%). When stratified by histology, germline P/LP variants were most frequently observed in serous carcinomas (8/28; 28.5%), while one endometrioid carcinoma (1/2; 50%) harbored a germline P/LP variant. No germline P/LP variants were detected in the small clear-cell or mucinous subgroups.

Statistical comparison between serous and non-serous tumors using Fisher’s exact test did not demonstrate a significant association between serous histology and the presence of germline P/LP variants (odds ratio [OR] = 2.67, *p* = 0.642). These findings suggest that, within this cohort, germline pathogenic variants are not confined to a single histological subtype, although their predominance in serous carcinomas reflects the overall distribution of histologies in the study population.

### Somatic variant distribution by histological subtype

Somatic alterations detected using the targeted solid-tumor panel showed clear histotype-related trends. Somatic TP53 variants were the most frequent alterations overall and were predominantly identified in serous carcinomas (18/28; 69.2%). In contrast, TP53 variants were observed in only one endometrioid case and one clear-cell case. Fisher’s exact testing comparing serous versus non-serous tumors indicated a higher likelihood of TP53 alterations in serous carcinomas (OR = 3.0), although this did not reach statistical significance (*p* = 0.377), likely reflecting the limited number of non-serous cases.

Other somatic alterations demonstrated more heterogeneous distributions. PIK3CA variants were detected in both serous and non-serous tumors (2 serous cases, 1 endometrioid and 1 clear-cell case), with no significant association with serous histology (OR = 0.21, *p* = 0.190). KRAS mutations were identified in one serous and one endometrioid carcinoma, while single cases of BRAF and ERBB2 alterations were observed exclusively within the serous subgroup. One patient without available histological classification harbored an AKT1 pathogenic variant. Although formal statistical testing was limited by low event counts, these findings are consistent with known subtype-specific molecular heterogeneity in ovarian cancer.

### Combined germline–somatic patterns within histological subtypes

Integration of germline and somatic findings revealed that several patients with serous carcinoma carried both germline susceptibility variants and somatic driver alterations, most commonly involving germline defects in homologous recombination–related genes together with somatic TP53 mutations. This combined pattern was not observed in the small number of non-serous tumors with germline P/LP variants, underscoring the molecular complexity and potential clinical relevance of dual-layer genomic profiling, particularly in serous ovarian cancer.

### Age and family history in relation to histology and germline status

Median age at diagnosis did not differ significantly across histological subtypes (Kruskal–Wallis test, *p* = 0.613). However, patients carrying germline P/LP variants tended to be diagnosed at a younger age compared with non-carriers (median 52 vs. 61 years), although this difference did not reach statistical significance (Mann–Whitney U test, *p* = 0.065).

In contrast, a strong association was observed between reported family history of cancer and the presence of germline P/LP variants. Patients with a positive family history were significantly more likely to harbor a germline pathogenic variant than those without such history (OR = 8.8, *p* = 0.034), irrespective of histological subtype. This finding supports the clinical relevance of family history as a predictor of hereditary cancer risk, while also highlighting that germline variants may still be present in patients without an overt familial burden.

### Stratification of serous carcinomas by tumor grade

Within the serous carcinoma subgroup (*n* = 28), tumors were further stratified by histological grade into high-grade serous (HGSC; G3, *n* = 23) and low-grade serous carcinomas (LGSC; G1–G2, *n* = 5).

### Germline variants in high-grade versus low-grade serous carcinomas

Germline P/LP variants were detected in 7 of 23 high-grade serous tumors (30.4%) and in 1 of 5 low-grade serous tumors (20%). Fisher’s exact test did not demonstrate a statistically significant difference between high-grade and low-grade serous carcinomas (OR = 2.69, *p* = 0.628), although a numerical enrichment of germline variants in high-grade tumors was observed.

### Somatic variants in high-grade versus low-grade serous carcinomas

Somatic TP53 alterations were more frequent in high-grade serous carcinomas (15/23; 65.2%) compared with low-grade serous carcinomas (3/5; 60.0%). This difference did not reach statistical significance (OR = 3.0, *p* = 0.330), but is consistent with the established role of TP53 as a defining molecular hallmark of high-grade serous ovarian cancer.

Other somatic alterations were infrequent and showed no statistically significant grade-dependent differences. PIK3CA variants were detected in one high-grade and one low-grade serous tumor (OR = 0.26, *p* = 0.415). KRAS and BRAF alterations were each observed in a single high-grade serous tumor and were absent in low-grade serous tumors (*p* = 1.0 for both comparisons). ERBB2 alterations were identified in one low-grade serous carcinoma but not in high-grade tumors (*p* = 0.231). These findings reflect the molecular heterogeneity of serous ovarian cancer and the limited power for detecting grade-specific associations in small subgroups.

### Age distribution in high-grade versus low-grade serous carcinomas

Median age at diagnosis was 60.5 years for patients with high-grade serous carcinoma and 55.0 years for those with low-grade serous carcinoma. The difference was not statistically significant (Mann–Whitney U test, *p* = 0.180).

## Discussion

This study provides a comprehensive molecular and clinical profile of 33 women diagnosed with ovarian carcinoma. The analysis encompassed both germline DNA, used for hereditary risk assessment, and tumor-derived DNA, relevant for prognostic stratification and therapeutic guidance, thereby offering a dual-layered perspective on inherited susceptibility and somatic mutational patterns. High-grade serous carcinoma (HGSC) was the predominant histological subtype in the cohort, in line with global epidemiological trends [1, 2].

The frequency of germline pathogenic and likely pathogenic variants observed in the present cohort (27.3%) falls within the range reported in large international studies, where hereditary predisposition is identified in approximately 20–30% of ovarian cancer patients. These findings indicate that the germline variant burden detected in our cohort is not unexpected and is broadly consistent with estimates derived from unselected ovarian cancer populations [13].

At the same time, population-specific data on germline variant frequencies in Bulgarian ovarian cancer patients remain limited. In this context, our results provide novel insight into the hereditary genetic landscape of ovarian cancer in Bulgaria and contribute valuable population-level evidence that complements existing international datasets. The concordance with published estimates supports the generalizability of current genetic testing recommendations, while also underscoring the need for larger, nationally representative studies to refine population-specific risk assessment.

Germline pathogenic variants in high-penetrance genes such as BRCA1 and BRCA2 were identified in multiple cases, supporting previous observations that approximately 20–25% of ovarian cancers have a hereditary origin [3, 4]. In addition, pathogenic variants in moderate-penetrance genes, including RAD51D, FANCL, ATM, and NBN, were observed, highlighting the expanding spectrum of clinically relevant susceptibility genes incorporated into contemporary genetic testing strategies [5, 6]. Rare variants in the WRN gene were also detected, warranting further investigation into their potential modifier roles in ovarian carcinogenesis.

On the somatic level, TP53 was the most frequently altered gene, consistent with its established role as a key driver mutation in serous ovarian carcinomas [7]. Co-occurrence of TP53 alterations with PIK3CA variants was observed within the serous subtype, aligning with known histotype-specific molecular patterns [8]. The TP53 variants identified included several recurrent pathogenic substitutions (e.g., p.Arg248Trp, p.Tyr220Cys, and p.His179Tyr), with variant allele frequencies ranging from subclonal to clonal levels, suggesting heterogeneity in the timing of mutational events during tumor evolution.

Analysis of germline–somatic correlations revealed that a subset of patients carried pathogenic germline variants alongside concurrent somatic driver mutations, reflecting complex tumor evolutionary trajectories. These findings underscore the importance of integrated genomic profiling in clinical decision-making and support the relevance of combined germline and somatic testing, particularly with respect to therapeutic strategies such as PARP inhibition in BRCA1/2-mutant or homologous recombination–deficient tumors [9, 10].

Within the present cohort, a positive family history of cancer was reported in 8 of 33 patients (24.2%), indicating a substantial proportion of individuals with a potential hereditary background. Statistical analysis demonstrated a significant association between reported familial cancer burden and the presence of germline pathogenic or likely pathogenic variants. Patients with a positive family history were significantly more likely to harbor a germline pathogenic variant than those without such history (odds ratio = 8.8, *p* = 0.034).

This observation reinforces the continued clinical relevance of family history as an indicator of hereditary cancer susceptibility in ovarian cancer. In several cases, positive family history coincided with pathogenic variants in high- or moderate-penetrance genes involved in DNA repair and homologous recombination pathways, supporting the biological plausibility of this association. Similar findings have been reported in large cohort studies demonstrating enrichment of germline BRCA1/2 and other homologous recombination repair gene variants among ovarian cancer patients with familial cancer aggregation [13, 14].

Importantly, germline pathogenic variants were also identified in patients without a reported family history of cancer. This finding highlights a key limitation of genetic testing strategies based solely on family history. Factors such as small family size, incomplete or inaccurate family information, sex-limited inheritance patterns, and the presence of de novo or moderate-penetrance variants may obscure hereditary predisposition. Previous studies have similarly shown that a substantial proportion of ovarian cancer patients with germline pathogenic variants would not meet traditional family history–based testing criteria [14, 15]. These observations support broader or universal germline testing approaches in ovarian cancer, irrespective of reported familial cancer burden.

In contrast, no statistically significant association was observed between family history and the presence or pattern of somatic driver mutations. Somatic alterations, most notably TP53 mutations, were distributed independently of familial cancer status and were instead strongly associated with histological subtype and tumor grade. This finding is consistent with established molecular models of ovarian carcinogenesis, in which somatic TP53 mutations represent early and nearly ubiquitous events in high-grade serous carcinoma, regardless of inherited risk [16, 17].

Overall, these results emphasize the complementary roles of clinical family history and molecular profiling in ovarian cancer. While a positive family history remains a strong predictor of germline pathogenic variants, its absence does not exclude hereditary susceptibility. Integration of family history with germline genetic testing and somatic tumor profiling provides a more robust and equitable framework for hereditary risk assessment, genetic counseling, and personalized management strategies.

Stratification by histological subtype demonstrated that serous carcinomas accounted for the majority of both germline and somatic alterations in this cohort, largely reflecting their predominance among enrolled patients. Germline pathogenic variants were distributed across histological subtypes without statistically significant restriction. In contrast, somatic TP53 alterations showed clear enrichment in serous tumors, particularly within the high-grade subgroup, consistent with established biological models of ovarian carcinogenesis. Although most associations did not reach statistical significance due to limited sample size, the observed trends support the relevance of histology- and grade-specific genomic stratification in ovarian cancer [16, 18, 19].

Beyond PARP inhibition, the somatic variants identified in this cohort suggest potential, albeit exploratory, avenues for personalized therapeutic consideration. Alterations in genes such as PIK3CA, KRAS, ERBB2, and AKT1 have been associated with targeted or pathway-directed therapies in other solid tumors and are currently being evaluated in ovarian cancer within clinical trial or investigational settings [16, 20, 21]. Although these alterations are not currently considered standard-of-care predictive biomarkers in ovarian cancer, their detection reflects the expanding molecular taxonomy of the disease and the growing interest in histology-agnostic and pathway-driven treatment strategies.

It should be noted that the targeted design of the somatic panel used in this study does not allow comprehensive interrogation of all actionable pathways, and these findings should therefore be interpreted with caution. Nevertheless, the presence of potentially targetable alterations supports the clinical value of broader molecular profiling and aligns with emerging precision oncology frameworks that aim to match selected ovarian cancer patients to targeted or basket-trial therapies beyond established PARP inhibitor indications [22, 23].

Finally, clinical characteristics such as age at diagnosis, reproductive history, and body mass index were consistent with known risk profiles for ovarian cancer. The youngest patient in the cohort (20 years of age) harbored a low-grade (G1) tumor without an identifiable germline variant, whereas the oldest patient (83 years) presented with high-grade serous carcinoma and a pathogenic TP53 mutation, illustrating the broad clinical and genomic heterogeneity of ovarian cancer.

Several limitations should be acknowledged. The relatively small cohort size limits statistical power, particularly for analyses involving rare histological subtypes and low-frequency somatic alterations. Importantly, although the study was designed to assess clinically actionable pathways, the use of targeted germline and somatic panels does not allow comprehensive evaluation of all molecular mechanisms relevant for therapy selection, such as full homologous recombination deficiency profiling or emerging biomarkers detectable by whole-exome or whole-genome sequencing.

## Conclusions

Dual-sample NGS profiling provides additional insights into the detection of clinically actionable variants in ovarian cancer. Our findings may support precision oncology strategies and hereditary risk assessment within the Bulgarian population.

## Data Availability

The datasets generated and/or analysed during the current study are available from the corresponding author on reasonable request.

## Abbreviations

NGS: Next‑generation sequencing
FFPE: Formalin‑fixed, paraffin‑embedded
HGSC: High‑grade serous carcinoma
VAF: Variant allele frequency
HR/HRR: Homologous recombination / homologous recombination repair
P/LP: Pathogenic / likely pathogenic
VUS: Variant of uncertain significance
ACMG/AMP: American college of medical genetics and genomics / association for molecular pathology
